# Off-Clamp Laparoscopic Partial Nephrectomy for a Fat-Poor Angiomyolipoma Arising from the Renal Capsule: A Case Report

**DOI:** 10.1155/2012/484790

**Published:** 2012-11-20

**Authors:** Koichi Kodama, Yasukazu Takase, Isamu Motoi, Katsuhiko Saito

**Affiliations:** ^1^Department of Urology, Toyama City Hospital, Toyama 939-8511, Japan; ^2^Department of Pathology, Toyama City Hospital, Toyama 939-8511, Japan

## Abstract

Renal function can be significantly preserved after nephron-sparing surgery by decreasing the intraoperative ischemic duration or by performing off-clamp surgery. We report the case of a 56-year-old woman diagnosed with a minimal-fat angiomyolipoma arising from the renal capsule, which was successfully treated by retroperitoneoscopic partial nephrectomy without hilar clamping. Computed tomography revealed a 16 × 13 mm homogenous lenticular mass protruding from the lateral aspect of the left kidney. On both T1- and T2-weighted magnetic resonance images, the mass exhibited homogenous low-signal intensity and well-defined margins. Laparoscopic magnification indicated that the exophytic tumor was attached to the renal cortex by a small peduncle. The tumor was resected completely with negative surgical margin. The estimated glomerular filtration rate after surgery was nearly equal to that before surgery. Off-clamp laparoscopic partial nephrectomy is a feasible surgical option to prevent ischemic renal damage in select patients presenting with small, exophytic, and peripheral renal masses.

## 1. Introduction

Angiomyolipoma (AML) is the most common benign tumor of the kidney, and it comprises variable amounts of fat, smooth muscle, and abnormal blood vessels. Most AMLs can be easily diagnosed by imaging because of their fat component, which increases signal intensity relative to the renal parenchyma [1]. In approximately 4.5% of all AML cases, however, the intratumoral fat content is low or obscured by mesenchymal elements, resulting in isointense images that may not assist in diagnosis [2, 3]. Here we report the case of a 56-year-old woman with a fat-poor AML arising from the renal capsule, which was successfully treated by off-clamp retroperitoneoscopic partial nephrectomy.

## 2. Case Presentation

A 56-year-old woman was referred to our institution for further investigation of a renal mass that was incidentally detected by computed tomography (CT). The patient had no obvious symptoms. She was afebrile, and findings of physical examination, routine laboratory tests, and urinalysis were unremarkable.

**Figure 1:**
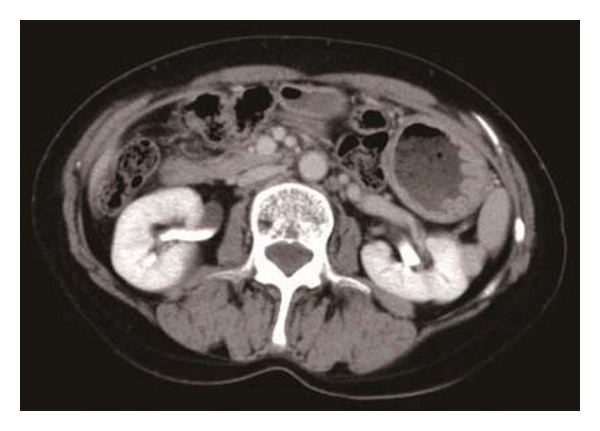
Contrast-enhanced computed tomography showing a homogenously enhanced lenticular tumor from the lateral aspect of the middle pole of the left kidney.

**Figure 2:**
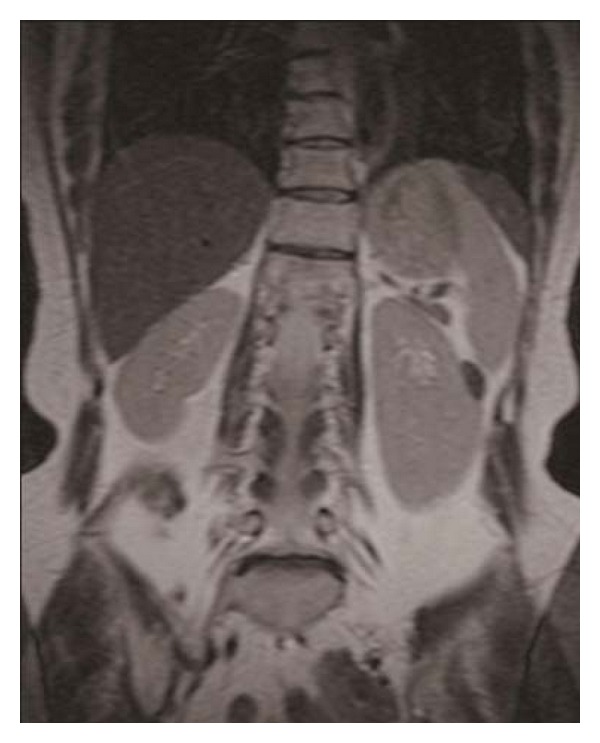
Coronal T1-weighted magnetic resonance imaging showing a tumor with homogenous low-signal intensity and well-defined margins.

Unenhanced two-dimensional CT revealed a 16 × 13 mm homogenous lenticular mass protruding from the lateral aspect of the middle pole of the left kidney. The tumor was isodense compared with the healthy renal parenchyma; therefore, it was imaged by contrast-enhanced CT, which revealed a homogeneous mass with well-defined margins and a smooth interface between the mass and kidney (Figure 1). On both T1- and T2-weighted magnetic resonance (MR) images, the tumor exhibited homogeneous low-signal intensity and well-defined margins (Figure 2), thus appearing benign. In addition, it seemed to originate from the renal capsule.

**Figure 3:**
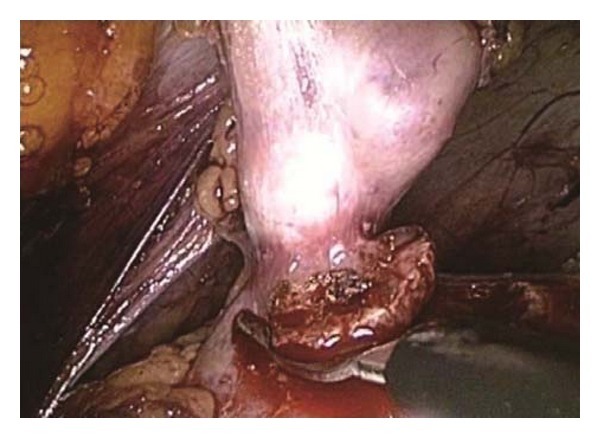
Retroperitoneoscopic view showing tumor excision without hilar clamping. The tumor is attached to the renal cortex by a small peduncle.

Retroperitoneoscopic partial nephrectomy was performed with the patient in the full-flank position. After a retroperitoneal dissection balloon was inflated to expand the retroperitoneal space, three laparoscopic ports (12, 12, and 5 mm) were inserted. The kidney, covered by Gerota's fascia, was identified and the renal hilum was dissected. A vascular tape was introduced through the port and passed under the renal artery in case bulldog clamping was required. The kidney was mobilized within Gerota's fascia to identify the mass. Laparoscopic magnification indicated that the exophytic tumor was attached to the renal cortex by a small peduncle. The tumor was excised through the renal cortex using cold scissors (Figure 3). Complete resection with negative surgical margins was achieved. The fat overlying the mass was not dissected and was removed as part of the pathological specimen. The tumor bed was virtually free of blood, although severe bleeding may occur from the renal parenchymal margin during off-clamp partial nephrectomy. We placed Avitene (microfibrillar collagen, Alcon, Inc., Humacao, PR) on the parenchymal surgical margin to accelerate hemostasis. The patient did not require renal parenchymal suture repair, hilar clamping, or drainage. The total surgical duration was 170 minutes, and the intraoperative blood loss was 10 mL.

**Figure 4:**
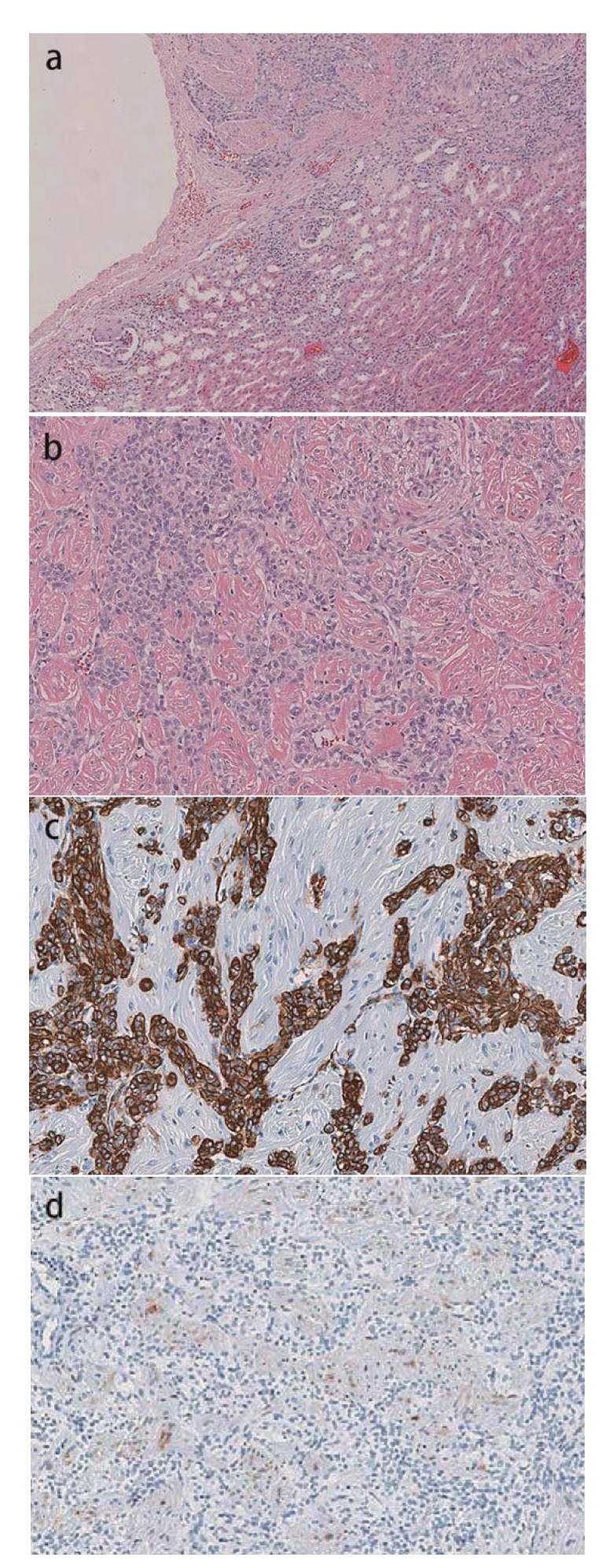
Histopathological examination. (a) The tumor is contiguous with the renal capsule (H&E staining, ×40). (b) Both epitheloid and spindle cells proliferate in an interlacing fascicular arrangement. No fat cells are present (H&E staining, ×100). (c) The epitheloid cells are diffusely and strongly positive for *α*-smooth muscle actin (×100). (d) The spindle cells are focally and weakly positive for HMB-45 (×100).

A cross-section of the surgical specimen revealed a gray-tan solid tumor measuring 21 × 18 × 10 mm. The tumor was contiguous with the renal capsule and did not extend into the perirenal adipose tissue (Figure 4(a)). Histological examination revealed both epitheloid and spindle cells proliferated in an interlacing fascicular arrangement without incorporating renal tubules (Figure 4(b)). Individual tumor cells contained ovoid or cleaved nuclei, abundant clear cytoplasm, and copious glycogen. No fat cells were found. Immunohistochemical analysis revealed that the epitheloid cells were diffusely and strongly positive for *α*-smooth muscle actin (Figure 4(c)), whereas the spindle cells were focally and weakly positive for HMB-45 (Figure 4(d)). The tumor cells were negative for S100 protein. A final diagnosis of fat-poor AML arising from the renal capsule was made.

There was no evidence of acute or delayed renal hemorrhage and urine leakage. Pre- and postoperative estimated glomerular filtration rates (eGFRs) were both 83.5 mL/min/1.73 m^2^, and the patient was discharged 6 days after surgery. There were no signs of recurrence on follow-up CT performed 6 months after surgery.

## 3. Discussion

Renal AML usually manifests as an exophytic growth, with most of the neoplasm often extending into the perirenal space. Large exophytic AMLs and well-differentiated retroperitoneal liposarcomas, which are often closely associated with the renal capsule, may have similar appearances on CT images. For a typical AML, however, a defect in the renal parenchyma at the tumor origin will be observed on CT because AMLs usually arise from within the kidney. Therefore, careful evaluation of CT images should distinguish AMLs from liposarcomas [4]. In the present case, however, the tumor arose from the renal capsule without invasion into the adjacent renal parenchyma, which gave the appearance of a smooth interface between the lesion and kidney on MR images. Moreover, the fact that the tumor was small and included no fat component made definitive preoperative diagnosis of AML difficult by CT and MR imaging only.

Renal ischemia-reperfusion injury caused by vascular clamping contributes to the decline in eGFR following partial nephrectomy. In 800 patients undergoing laparoscopic partial nephrectomy, functional outcome as measured by the postoperative decrease in eGFR was significantly better after surgery with shorter ischemic duration (<20 min, 9% decrease) than after surgery with longer ischemic duration (20–30 min of ischemia, 15% decrease; >30 min of ischemia, 23% decrease) [5]. In a review of 362 patients with a solitary kidney undergoing partial nephrectomy, each additional minute of ischemia significantly increased the risk of acute kidney injury, early postoperative eGFR <15 mL/min/1.73 m^2^, and onset of stage 4 chronic kidney disease [6].

Recent reports advocating partial clamping, early unclamping, and no clamping during laparoscopic partial nephrectomy have demonstrated technical modifications that may further aid in the preservation of postoperative renal function. In a series of patients with tumors involving a solitary kidney, the preservation of renal function was 33% better with off-clamp partial nephrectomy than with complete hilar control partial nephrectomy [7]. Off-clamp surgery can also be used to treat larger and more complex renal tumors without altering the surgical duration, blood loss, hospital stay, complication rate, and positive surgical margin rate, unlike complete hilar control clamping [8]. However, it should be emphasized that severe bleeding may occur from the renal parenchymal margin during off-clamp partial nephrectomy. Additional cases and longer followups are necessary to confirm the preservation of renal function after off-clamp partial nephrectomy.

Fat-poor AML may be difficult to diagnose by imaging only, especially when the mass arises from the renal capsule rather than the renal parenchyma. Off-clamp laparoscopic partial nephrectomy may avoid renal damage and preserve renal function after surgery in select patients with small, exophytic, and peripherally located renal masses.
